# Evaluation of severe and chronic factors for extended stays in Japanese medical treatment and supervision act wards

**DOI:** 10.3389/fpsyt.2025.1653427

**Published:** 2025-11-27

**Authors:** Naoya Takeda, Hiroko Kashiwagi, Norio Watanabe, Naotsugu Hirabayashi

**Affiliations:** 1 Department of Forensic Psychiatry, National Center Hospital, National Center of Neurology and Psychiatry, Tokyo, Japan; 2 Translational Medical Center, National Center of Neurology and Psychiatry, Tokyo, Japan

**Keywords:** forensic psychiatry, severe and chronic criteria, psychiatric symptoms, behavioral disorders, life disorders, length of hospitalization

## Abstract

**Objective:**

The Japanese Ministry of Health, Labour and Welfare introduced the “severe and chronic” criteria to describe conditions associated with long-term psychiatric hospitalization. This study examined factors contributing to prolonged hospitalization in Medical Treatment and Supervision (MTS) Act wards using these criteria.

**Methods:**

The “severe and chronic” criteria comprise three components: “Psychiatric symptoms,” “Behavioral disorders,” and “Life disorders”. As of January 10, 2014, 210 patients hospitalized in MTS Act wards for 1.5 years were enrolled. Treatment outcomes were assessed at 1.5, 2.5, and 3.5 years, and associations between hospitalization duration and each criterion were analyzed.

**Results:**

By 3.5 years, 185 patients had been discharged. At this timepoint, “Psychiatric symptoms” and “Life disorders” were significantly associated with hospitalization outcomes. Twenty individual items—primarily subitems of “Psychiatric symptoms” and “Life disorders”—were consistently related to hospitalization at all timepoints (p < 0.05).

**Conclusions:**

The “Psychiatric symptoms” and “Life disorders” components of the “severe and chronic” criteria were significantly associated with hospitalization outcomes in MTS Act wards.

## 1 Introduction

Forensic mental health facilities are being established globally to improve the mental condition of offenders with mental disorders and reduce recidivism risk. In Japan, the judicial mental health system was reformed in 2005 with the enactment of the “Act on Medical Treatment and Observation for Persons Who Have Seriously Injured Others While in a State of Insanity,” commonly referred to as the Medical Treatment and Supervision (MTS) Act (1). Under this system, individuals who commit serious offenses while in a state of insanity or diminished capacity are managed through a designated judicial framework. During these proceedings, the prosecutor files a motion, and the district court issues a treatment decision. The panel comprises one judge and one psychiatrist holding a national license as a mental health examiner.

The panel may issue one of three decisions: admission to an MTS Act ward for inpatient treatment, outpatient treatment in the community, or exclusion from the MTS system. Offenders ordered into the system receive specialized psychiatric care in designated medical institutions and are continuously monitored by rehabilitation coordinators based in probation offices. This framework allows individuals who committed offenses while mentally ill to receive coordinated treatment under the jurisdiction of both the courts and the Ministry of Justice, marking a new era in Japanese forensic mental health services (2).

Since then, Japan’s MTS medical observation system has operated for approximately 17 years and is becoming an established component of psychiatric care. The system is characterized by structured rehabilitation processes and inter-agency collaboration that aim to reintegrate offenders into the community while reducing recidivism (3). It provides active treatment through multi-professional and multi-institutional collaboration, promoting the social rehabilitation of individuals with mental disorders who committed serious offenses while in a state of insanity or diminished capacity (4–6). The MTS Act wards are divided into units for acute, recovery, and social reintegration, and shared care (for women), and patients move between units as their treatment progresses. This clarifies the patients and treatment goals in each unit, and allows for the provision of an environment and treatment content suited to each treatment stage.

The MTS Act treatment guidelines divide hospitalization into three phases: acute (3 months), recovery (9 months), and social reintegration (6 months), with specific goals for each stage. The intended duration of hospitalization is approximately 18 months. This framework was originally established by the MHLW (7). In the acute phase, objectives include improving psychopathological symptoms and mental status, promoting physical recovery and psychological stability, confirming treatment motivation, and establishing a therapeutic alliance. During the recovery phase, goals focus on enhancing illness insight and self-control, restoring daily living skills through participation in structured treatment programs, and achieving clinical stability sufficient for supervised walks and brief outings. In the social reintegration phase, aims include maintaining stability to permit extended outings and overnight stays, fostering acceptance of disability through therapeutic engagement, restoring independent living skills such as medication adherence and financial management, and preparing for community reintegration (8). These objectives were defined by the MHLW (8).

However, as of July 15, 2015, 2,564 individuals hospitalized in MTS Act wards had an average stay of 940 days (median: 758 days), equivalent to approximately two years. Nearly 20% of patients had been hospitalized for three years or more—double the standard hospitalization period—highlighting a critical issue in Japan’s medical observation system (9).

Prolonged hospitalization in forensic psychiatric facilities is a widespread issue globally (10, 11). In the United Kingdom, extended hospital stays often result from delays in reducing security levels and repeated transfers between high- and medium-security hospitals. Although progress is being made in understanding the outcome of personality disorders, and it is now known that self-mutilation and help-seeking suicide threats or attempts resolve relatively quickly (12), factors such as comorbid personality disorders, histories of self-harm or violence, and challenges in treatment responsiveness during psychiatric care also contribute to prolonged hospitalization (13). Identifying factors associated with extended stays and preventing unnecessary prolongation are essential to support successful social reintegration. Internationally, studies have identified several contributing factors to extended hospital stays in forensic settings, such as lack of community placement options, high security needs, and inadequate discharge planning. In England, a mixed-methods study found that long-stay patients often remained institutionalized not due to active clinical risk but because of systemic issues such as limited inter-unit coordination and inconsistent discharge criteria (11, 13). For instance, in the United Kingdom, system-level barriers, such as difficulties in transferring between secure units, have been cited as major contributors to long stays (13). Additionally, comorbid substance use, treatment resistance, and legal complexities are recurrent themes in Europe and North America (10, 11).

**Figure 1:**
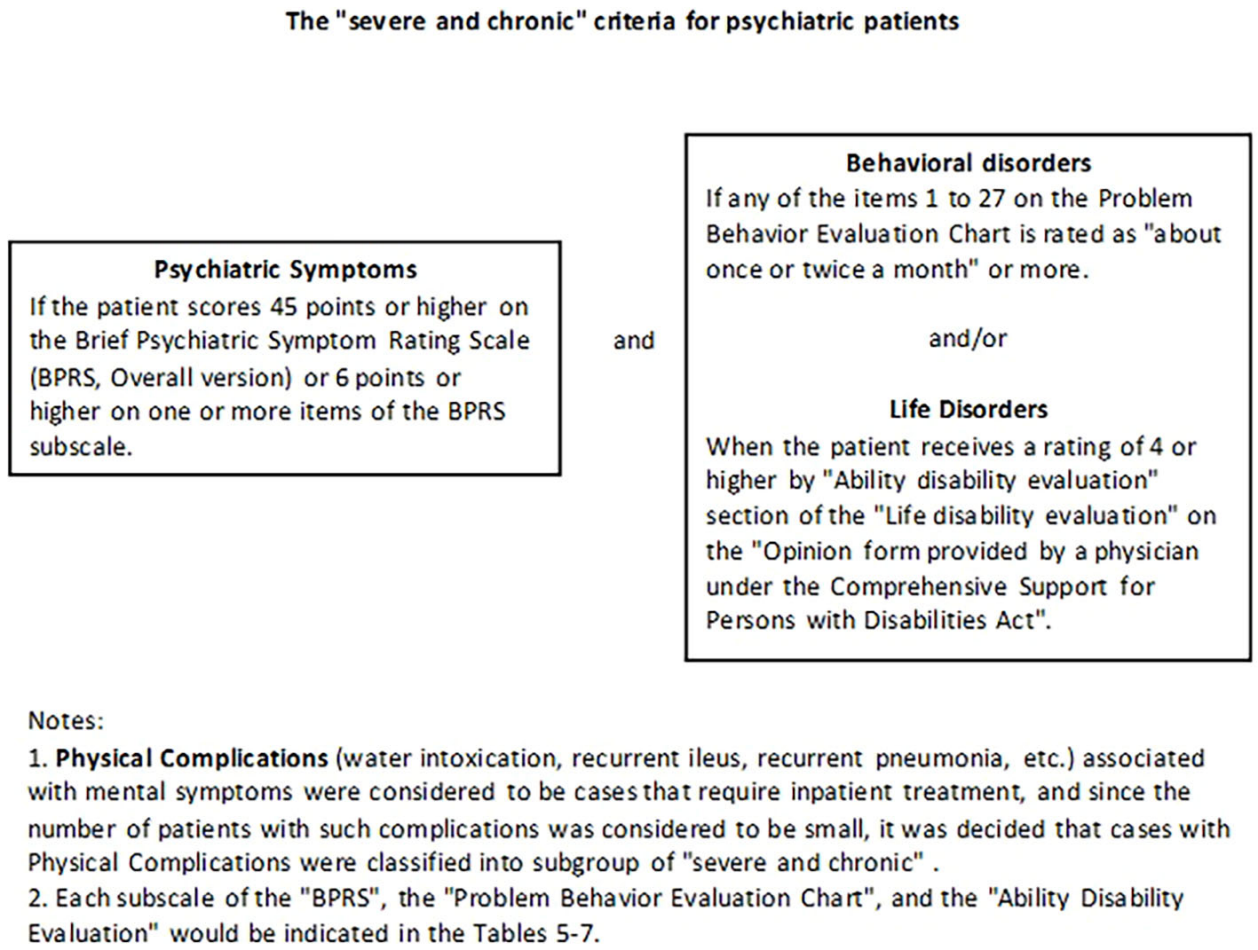
Proposed “severe and chronic” criteria for psychiatric patients. “Physical complications” refer to cases that require inpatient treatment. These cases are classified into the “severe and chronic” subgroups.

Long-term hospitalization for schizophrenia—a condition that accounts for most patients in MTS Act wards—is also a global concern in general psychiatry. Identifying factors contributing to prolonged hospitalization in individuals with schizophrenia is essential to prevent unnecessary institutionalization (14–17). As of March 31, 2013, a provisional standard for defining “severe and chronic” cases in Mental Health and Welfare Law wards was developed by the Comprehensive Research Project for Measures for Persons with Disabilities, funded by research grants from the MHLW (18). This standard focuses on patients with chronic disease progression, high clinical severity, behavioral pathology, or treatment resistance, rather than those who remain hospitalized due to a lack of community support. In other words, the interim criteria target clinical factors underlying long-term hospitalization, excluding so-called “social hospitalization”. As of March 31, 2016, “physical complications” were removed from consideration, and a revised criterion was developed to reflect this change (**Figure 1**; 19).

Given the shared aim of facilitating rehabilitation and preventing chronic institutionalization, these criteria may also be relevant to MTS Act wards. However, their applicability to forensic psychiatric settings has not been empirically examined yet. To identify factors contributing to long-term hospitalization for patients in MTS Act wards, this study examined all patients hospitalized for 1.5 years or more (the standard maximum hospitalization period set by the Ministry of Health, Labour and Welfare) in 32 MTS Act wards in Japan as of January 10, 2014, to determine whether they met the “severe and chronic” criteria 1.5, 2.5, and 3.5 years after enrollment. The large-scale survey was only examined by a research group associated with MHLW and has not yet been published. This study was conducted by a research group associated with MHLW, and the authors were given permission to conduct their analysis.

## 2 Materials and methods

### 2.1 Subjects

An initial survey was conducted on January 10, 2014, targeting patients hospitalized for 1.5 years or longer in 32 designated inpatient facilities nationwide, established under the MTS framework designated by the MHLW. Of the 791 patients hospitalized in MTS Act wards on that date, 563 had been hospitalized for less than 1.5 years and were excluded. Among the remaining 228, two declined participation after being informed about the study, resulting in an inclusion rate of 99.1%. After excluding 10 patients with unknown outcomes and six who were transferred to other hospitals, a final total of 210 patients were enrolled.

### 2.2 Methods

This prospective cohort study examined patients hospitalized under the MTS Act for 1.5 years or longer, using data from 32 designated institutions nationwide. Cox regression and descriptive statistics were used for analysis. In 2015, the MHLW Research Group established criteria for identifying “severe and chronic” psychiatric patients (**Figure 1**). These criteria include four components: “Psychiatric symptoms,” “Behavioral disorders,” “Life disorders,” and “Physical complications (18)”. To distinguish “Psychiatric symptoms” as a specific criterion for “severe and chronic” status — rather than general psychiatric symptoms — we enclosed the term in quotation marks (18).

#### 2.2.1 Psychiatric symptoms

Psychiatric symptoms were assessed using the Brief Psychiatric Rating Scale (BPRS), a widely-validated instrument for evaluating the severity of psychiatric symptoms. The BPRS consists of 18 items: conceptual disorganization, grandiosity, hostility, suspiciousness, hallucinatory behavior, excitement, emotional withdrawal, blunted affect, somatic concern, anxiety, guilt feelings, tension, mannerisms and posturing, depressive mood, motor retardation, uncooperativeness, unusual thought content, and disorientation.

Patients scoring ≥45 on the Overall version of the BPRS or ≥6 on any individual item from the subscale were classified as having “psychiatric symptoms”. Positive symptom scores were calculated by summing the scores for conceptual disorganization, grandiosity, hostility, suspiciousness, hallucinatory behavior, and excitement. Negative symptom scores comprised emotional withdrawal and blunted affect. The comprehensive psychopathology score included the remaining ten items: somatic concern, anxiety, guilt feelings, tension, mannerisms and posturing, depressive mood, motor retardation, uncooperativeness, unusual thought content, and disorientation. These subscale scores, along with the total BPRS score, were included in the analysis.

#### 2.2.2 Behavioral disorders

Behavioral disorders were assessed using 27 items: suicidal ideation, suicidal behavior, verbal outbursts, physical violence, impulsiveness, property damage, seeking others, sexual acts, arson, incontinence, mysophilia, disrobing, collectomania, epileptic seizures, irritability, poor concentration, fixation on specific objects or individuals, stress intolerance, medication non-compliance, refusal to assist, wandering, hyperactivity or inactivity, compulsions, appetite changes, polydipsia, pica, and substance use. These items, derived from the Problem Behavior Evaluation Chart, represent clinical behaviors commonly observed in long-term psychiatric inpatients. Notably, some items—such as poor concentration—may reflect cognitive impairment rather than discrete behavioral disturbances. Prior research has linked such impairments with treatment resistance and functional decline, which may indirectly contribute to prolonged hospitalization. Patients were classified as having behavioral disorders if they scored ≥1, indicating the behavior occurred “about once or twice a month”. According to the provisional criteria for “severe and chronic” status, two subscores were calculated: an A score, related to self-harm and harm to others (suicidal ideation, suicidal behavior, verbal outbursts, physical violence, impulsiveness, property damage, seeking others, sexual acts, and arson); and a B score, encompassing the remaining items. Both subscores, along with the presence of behaviors occurring at least once or twice a month, were included in the analysis.

#### 2.2.3 Life disorders

Based on the official medical certificate for persons with disabilities in Japan, functional status was assessed using 15 items: mobility in bed, transferring, eating, continence, bathing, dressing and undressing, meal preparation, daily living rhythm, hygiene, financial management, medication use, interpersonal relationships, telephoning, shopping, and transportation. These items were adapted from the Ability Disability Evaluation and align with standard indicators of functioning under Japan’s disability framework. Their relevance to social integration and rehabilitation planning has been supported by prior research, highlighting their utility in evaluating discharge readiness. A score of 4 or higher on the Ability Disability Evaluation was considered indicative of substantial support needs in daily functioning. Based on clinical guidance from the Act on Support and Support for Persons with Disabilities, subscores were calculated as follows: the behavioral disabilities score included mobility in bed, transferring, eating, continence, bathing, and dressing/undressing; the community life disorders score included meal preparation, living rhythm, hygiene, financial management, medication use, and interpersonal relationships, as presented in the 2007 National Welfare Office Directors Conference as core elements of community life; and the social adaptation interference score comprised telephoning, shopping, and transportation. These subscores, along with the threshold of daily life difficulty (score ≥4), were included in the analysis.

#### 2.2.4 Physical complications

Patients were considered to have physical complications if they presented with conditions such as water intoxication, recurrent ileus, or recurrent pneumonia during hospitalization in MTS Act wards. Physical comorbidities are common among individuals with mental disorders. Conversely, in Japan’s aging society, the incidence of mental illness — including depression and dementia — among older adults is notably high. Therefore, addressing the comorbidity of physical and psychiatric conditions is a critical concern. In recognition of this, “Physical complications” were added to the provisional criteria for identifying “severe and chronic” cases. Although such physical complications occur in only about 10% of inpatients and are generally secondary to psychiatric symptoms, they were classified as a distinct subgroup within the “severe and chronic” criteria. Importantly, no patients met the criteria for both “Psychiatric symptoms” and “Physical complications” without also meeting criteria for either “Behavioral disorders” or “Life disorders.” As a result, there was no difference in the total number of patients identified using the provisional versus the proposed “severe and chronic” criteria. Physicians at participating institutions assessed patients using the provisional “severe and chronic” criteria based on medical records. Scores were subsequently revised according to the proposed criteria. The primary survey collected data on age, sex, duration of hospitalization, psychiatric diagnosis, and treatment outcomes. Diagnoses were coded using the International Statistical Classification of Diseases and Related Health Problems, 10th Edition (ICD-10). Patient outcomes — categorized as discharged, still hospitalized, or transferred — were recorded at 1.5, 2.5, and 3.5 years using an outcome questionnaire. These outcomes reflect status as of the respective survey dates, rather than the specific discharge dates. July 15 was selected as the outcome survey date, as it marks the anniversary of the MTS Act’s implementation. The research group defined the 1.5-, 2.5-, and 3.5-year follow-ups as corresponding to more than 3, 4, and 5 years, respectively, since initial hospitalization.

### 2.3 Statistical analysis

Cox regression analysis was conducted to examine the association between clinical variables and treatment outcomes (discharged vs. inpatient) at 1.5, 2.5, and 3.5 years. The dependent variable was outcome status, while independent variables included age, sex, duration of hospitalization at the time of the survey, and the components of the proposed “severe and chronic” criteria (“Psychiatric symptoms,” “Behavioral disorders,” “Life disorders”) as well as “Physical complications”. Given the previously established association between psychiatric hospitalization duration and both age and sex in MTS Act wards, these variables were included as covariates in the analysis (20).

Additionally, multiple linear regression analysis was performed using the actual length of stay (in days) as the dependent variable. Independent variables included age, sex, psychiatric diagnosis, and the three primary components of the proposed “severe and chronic” criteria. This analysis was conducted to capture a more nuanced understanding of hospitalization duration, independent of policy-defined time points. However, despite repeated attempts at multiple regression analysis, there were missing values for 1.5-year timepoint.

To identify factors contributing to long-term hospitalization, we conducted Cox proportional hazards regression analysis using discharge outcome as the dependent variable. Independent variables included the components of the proposed criteria — “Psychiatric symptoms,” “Behavioral disorders,” and “Life disorders” — as well as “Physical complications,” their sub-items, and hospitalization duration. A hazard ratio significantly less than 1 was interpreted as indicative of a lower likelihood of discharge. For exploratory purposes, no Bonferroni correction was applied. Statistical significance was defined as p < 0.05.

Statistical analyses were conducted using SPSS for Windows, version 20.0 (IBM Corp., Armonk, NY).

### 2.4 Ethical approval

Ethical approval was obtained from the Ethics Committee of the National Center of Neurology and Psychiatry (Approval No. A2014-004; study title: Investigation of the Criteria for “Severe and Chronic” in Medical Observation Law Wards). The study was conducted in accordance with the latest revision of the Declaration of Helsinki. Written informed consent was obtained from all participants prior to enrollment.

## 3 Results

### 3.1 Subject summary

The 210 enrolled subjects had a mean hospitalization duration of 1,026 days (95% confidence interval [CI]: 974–1,079; median: 943 days). By July 15, 2015, 126 patients (60.0%) had been discharged; by July 15, 2016, 166 (79.0%); and by July 15, 2017, 185 (88.1%). As of the final survey, 25 patients (11.9%) remained hospitalized in MTS Act wards (**Table 1**). The median length of stay was 943 days at baseline, increasing to 1,516 days at 1.5 years, 1,950.5 days at 2.5 years, and 2,246 days at 3.5 years. Median lengths of stay were consistently longer for men than for women. Of the total sample, 171 patients (81.4%) were male, and 186 (88.6%) were diagnosed with ICD-10 F2 disorders (schizophrenia, schizophreniform disorder, or paranoid disorder). Among male patients, 11 (6.4%) were diagnosed with F1 disorders (mental and behavioral disorders due to psychoactive substance use), three (1.8%) with F0 disorders (organic, including symptomatic mental disorders), two (1.2%) with F3 disorders (mood disorders), one (0.6%) with an F4 disorder (neurotic, stress-related, and somatoform disorders), two (1.2%) with F8 disorders (developmental disorders), and one (0.6%) with a G40 condition (epilepsy). Among female patients, four (10.3%) were diagnosed with F3 disorders (**Table 2**). The mean age of the sample was 44 years (95% CI: 42–46 years). Regarding index offenses, 63 male cases (36.8%) involved injury, while 13 female cases (33.3%) involved attempted murder. The most common offenses were injury (34.8%), attempted murder (19.5%), arson (18.1%), and homicide (17.6%), followed by robbery (3.3%), indecent assault (3.3%), attempted arson (1.9%), and attempted robbery (1.4%). Robbery, indecent assault, and attempted arson were reported only among male patients (**Table 3**). In terms of physical complications, 15 patients had water intoxication, eight had recurrent ileus, four had recurrent pneumonia, and 15 had other physical complications. A total of 28 patients presented with at least one physical complication.

**Table 1:** Patient outcomes.

Outcome	Males n=171		Females n=39		Total n=210	
	“Severe and chronic” n=64	Total	“Severe and chronic” n=15	Total	“Severe and chronic” n=79	Total
1.5 years after hospitalization						
Discharged	29	103	4	23	33	126
Hospitalized (%)	35 (54.7)	68 (39.8)	11(73.3)	16 (41.0)	46 (58.2)	84 (40.0)
		Total		Total		Total
2.5 years after hospitalization						
Discharged	45	137	8	29	53	166
Hospitalized (%)	19 (29.7)	34 (19.9)	7 (46.7)	10 (25.6)	26 (32.9)	44 (21.0)
		Total		Total		Total
3.5 years after hospitalization						
Discharged	52	150	12	35	64	185
Hospitalized (%)	12 (18.8)	21 (12.3)	3 (20.0)	4 (10.3)	15 (19.0)	25 (11.9)

**Table 2:** Breakdown of gender and diagnosis.

Diagnosed with ICD-10	Males	Females	Total
	n (%)	n (%)	n (%)
F0	3 (1.8)	0 (0.0)	3 (1.4)
F1	11 (6.4)	0 (0.0)	11 (5.2)
F2	151 (88.3)	35 (89.7)	186 (88.6)
F3	2 (1.2)	4 (10.3)	6 (2.9)
F4	1 (0.6)	0 (0.0)	1 (0.5)
F5	0 (0.0)	0 (0.0)	0 (0.0)
F6	0 (0.0)	0 (0.0)	0 (0.0)
F7	0 (0.0)	0 (0.0)	0 (0.0)
F8	2 (1.2)	0 (0.0)	2 (1.0)
F9	0 (0.0)	0 (0.0)	0 (0.0)
G40	1 (0.6)	0 (0.0)	1 (0.5)
Total	171 (100.0)	39 (100.0)	210 (100.0)

**Table 3:** Breakdown of gender and illegal acts.

Offenses	Males	Females	Total
	n (%)	n (%)	n (%)
Murder	29 (17.0)	7 (17.9)	36 (17.1)
Attempted Murder	32 (18.7)	10 (25.6)	42 (20.0)
Arson	30 (17.5)	9 (23.1)	39 (18.6)
Attempted Arson	2 (1.2)	1 (2.6)	3 (1.4)
Assault	58 (33.9)	11 (28.2)	69 (32.9)
Robbery	7 (4.1)	0 (0.0)	7 (3.3)
Attempted Robbery	3 (1.8)	1 (2.6)	4 (1.9)
Indecent Assault	7 (4.1)	0 (0.0)	7 (3.3)
Unknown	3 (1.8)	0 (0.0)	3 (1.4)
Total	171 (100.0)	39 (100.0)	210 (100.0)

Seventy-nine subjects met the proposed “severe and chronic” criteria (**Table 1**). Under the proposed framework, “Physical complications” were treated as a subgroup of the “severe and chronic” classification and therefore were not included in the primary category. Among the 28 subjects with “Physical complications,” 16 also met the criteria for “Psychiatric symptoms”. Notably, no subject met criteria for both “Psychiatric symptoms” and “Physical complications” without also exhibiting “Behavioral disorders” or “Life disorders”. As a result, the total number of subjects classified as “severe and chronic” remained unchanged when transitioning from the provisional standard to the proposed criteria.

### 3.2 Subject outcomes and involvement of three items included in proposed “severe and chronic” criteria

**Table 4** presents the associations between discharge status (discharged vs. hospitalized) at 1.5, 2.5, and 3.5 years and the following variables: age, male sex, “Psychiatric symptoms,” “Behavioral disorders,” “Life disorders,” “Physical complications,” and hospitalization duration.

**Table 4:** Comprehensive analysis 1.5, 2.5, and 3.5 years after date of survey.

1.5 years after date of survey	B	P value	Significance level	Hazard ratio	95% CI	
Age	0.007	0.423		1.007	0.991	1.023
Male	-0.42	0.076		0.657	0.413	1.045
**Psychiatric symptoms (BPRS total ≥45 or 1 item ≥6)**	**-0.479**	**0.037**	*	**0.62**	**0.396**	**0.97**
Behavioral disorders (about once or twice a month or more in the Behavior Evaluation Chart)	0.055	0.78		1.056	0.72	1.55
**Life disorders (≥4 in Ability/Disability Evaluation)**	**-0.605**	**0.02**	*	**0.546**	**0.327**	**0.911**
Physical complication	0.223	0.424		1.25	0.724	2.158

2.5 years after date of survey	B	P value	Significance level	Hazard ratio	95% CI	
Age	0.005	0.491		1.005	0.991	1.019
Male	-0.357	0.09		0.7	0.463	1.058
**Psychiatric symptoms (BPRS total ≥45 or 1 item ≥6)**	**-0.424**	**0.036**	*	**0.655**	**0.441**	**0.972**
Behavioral disorders (about once or twice a month or more in the Behavior Evaluation Chart)	0.277	0.139		1.32	0.914	1.906
**Life disorders (≥4 in Ability/Disability Evaluation)**	**-0.553**	**0.011**	*	**0.575**	**0.376**	**0.88**
Physical complication	-0.089	0.725		0.915	0.556	1.504

3.5 years after date of survey	B	P value	Significance level	Hazard ratio	95% CI	
Age	0.007	0.3		1.007	0.994	1.021
**Male**	**-0.407**	**0.036**	*	**0.666**	**0.455**	**0.974**
**Psychiatric symptoms (BPRS total ≥45 or 1 item ≥6)**	**-0.438**	**0.02**	*	**0.645**	**0.447**	**0.933**
Behavioral disorders (About once or twice a month or more in the Behavior Evaluation Chart)	0.27	0.137		1.31	0.918	1.868
**Life disorders (≥4 in Ability/Disability Evaluation)**	**-0.452**	**0.021**	*	**0.636**	**0.434**	**0.933**
Physical complication	0.118	0.599		1.125	0.726	1.742

*p < 0.05.

B, estimated regression coefficient; BPRS, Brief Psychiatric Rating Scale; CI, confidence interval; *p < 0.05.

“Psychiatric symptoms” and “Life disorders” were significantly associated with continued hospitalization at 1.5 and 3.5 years (p < 0.05). At 2.5 years, only “Life disorders” remained significantly associated with ongoing hospitalization. In contrast, “Behavioral disorders,” Age, Sex, “Physical complications,” and length of hospitalization at baseline were not significantly associated with hospitalization status at any timepoint.

### 3.3 Subject outcomes and involvement of sub-items of proposed “severe and chronic” criteria

**Tables 5**–**8** and **Supplementary Tables 1 and 2** present the results of the Cox regression analysis for exploratory purposes, including estimated regression coefficients, hazard ratios, 95% CIs, p-values, and significance levels for discharge and continued hospitalization at 1.5, 2.5, and 3.5 years. Analyses were conducted for the sub-items of “Psychiatric symptoms,” “Behavioral disorders,” “Life disorders,” and “Physical complications”. Notably, among the “Behavioral disorders,” only suicidal ideation had a hazard ratio greater than 1 and was significantly associated with discharge. When comparing outcomes at 3.5 years with those at 1.5 and 2.5 years, the following sub-items were consistently associated with prolonged hospitalization across all timepoints (p < 0.05).

**Table 5:** Psychiatric symptoms (BPRS total ≥45 or 1 item ≥6) 3.5 years after the date of the survey.

Items	B	P value	Significance level	Hazard ratio	Lower limit	Upper limit
**Positive symptoms**	**-0.054**	**<0.001**	***	**0.947**	**0.921**	**0.974**
-**Conceptual disorganization**	**-0.194**	**<0.001**	***	**0.823**	**0.746**	**0.909**
-Grandiosity	-0.105	0.058		0.900	0.808	1.004
-Hostility	-0.091	0.105		0.913	0.817	1.019
-**Suspiciousness**	**-0.126**	**0.010**	*	**0.881**	**0.801**	**0.970**
-**Hallucinatory behavior**	**-0.110**	**0.024**	*	**0.896**	**0.814**	**0.985**
-Excitement	-0.116	0.061		0.890	0.788	1.005
Negative symptoms	-0.036	0.198		0.965	0.914	1.019
-Emotional withdrawal	-0.039	0.441		0.961	0.870	1.063
-Blunted affect	-0.084	0.104		0.920	0.831	1.018
Comprehensive psychopathology	-0.018	0.116		0.983	0.961	1.004
-Somatic concern	0.031	0.554		1.031	0.931	1.142
-Anxiety	0.019	0.711		0.981	0.887	1.805
-Guilt feelings	0.058	0.484		1.060	0.901	1.247
-Tension	-0.041	0.479		0.960	0.856	1.076
-Mannerisms and posturing	-0.110	0.062		0.895	0.797	1.006
-**Depressive mood**	**0.173**	**0.037**	*	**1.189**	**1.011**	**1.399**
-Motor retardation	-0.012	0.829		0.988	0.887	1.100
-**Uncooperativeness**	**-0.149**	**0.008**	**	**0.862**	**0.773**	**0.961**
-**Unusual thought content**	**-0.126**	**0.004**	**	**0.882**	**0.810**	**0.960**
-Disorientation	-0.050	0.640		0.951	0.771	1.173
**Total**	**-0.017**	**0.005**	**	**0.983**	**0.971**	**0.995**

*p < 0.05; **p < 0.01; ***p < 0.001.

B, estimated regression coefficient; BPRS, Brief Psychiatric Rating Scale.

**Table 6:** Behavioral disorders (about once or twice a month or more in the Behavior Evaluation Chart) 3.5 years after the date of the survey.

Items	B	P value	Significance level	Hazard ratio	Lower limit	Upper limit
Total score of A items	-0.023	0.312		0.977	0.934	1.022
-Suicidal ideation	0.134	0.067		1.143	0.991	1.319
-Suicidal behavior	0.206	0.115		1.229	0.951	1.588
-**Verbal outbursts**	**-0.215**	**0.011**	*	**0.807**	**0.683**	**0.952**
-Physical violence	-0.218	0.139		0.804	0.602	1.074
-Impulsiveness	-0.107	0.212		0.899	0.761	1.062
-Damaging property	-0.11	0.44		0.896	0.678	1.184
-Seeks others	-0.089	0.361		0.915	0.756	1.107
-Sexual acts	-0.121	0.43		0.886	0.655	1.197
-Arson	0.16	0.408		1.174	0.803	1.716
Total score of B items	-0.016	0.073		0.984	0.967	1.001
-Incontinence	-0.155	0.177		0.857	0.684	1.072
-Mysophilia	-0.209	0.084		0.812	0.641	1.028
-Tear/take off clothing	-0.174	0.34		0.84	0.587	1.201
-Collectomania	-0.115	0.478		0.892	0.65	1.224
-Epilepsy seizures	0.265	0.467		1.303	0.639	2.66
-Irritability	-0.042	0.467		0.959	0.856	1.074
-Poor concentration	-0.035	0.384		0.965	0.892	1.045
-Strong commitment to specific things and people	-0.062	0.17		0.94	0.859	1.027
-**Resistance to stress**	**-0.117**	**0.013**	*	**0.889**	**0.811**	**0.975**
-Non-compliance with medication	0.042	0.625		1.043	0.882	1.232
-Refusal to assist	-0.098	0.206		0.907	0.779	1.055
-Wandering	-0.006	0.939		0.994	0.841	1.174
-Hyperactivity/suspension of action	-0.032	0.639		0.968	0.847	1.107
-Compulsions	-0.081	0.172		0.922	0.82	1.036
-Increased/decreased appetite	0.109	0.275		1.115	0.917	1.355
-Polydipsia	-0.075	0.216		0.927	0.823	1.045
-Pica	-0.039	0.858		0.962	0.626	1.478
-**Substance use**	**0.513**	**0.017**	*	**1.67**	**1.097**	**2.542**
Probl+H15:M35ematic behavior ≥1–2 times a month	-0.067	0.674		0.935	0.684	1.278

*p < 0.05.

B, estimated regression coefficient.

**Table 7:** Life disorders (≥4 in ability/disability evaluation) at 3.5 years after the date of the survey.

Items	B	P value	Significance level	Hazard ratio	Lower limit	Upper limit
Behavioral disabilities	0.001	0.969		1.001	0.943	1.063
-Mobility on the bed	0.209	0.282		1.233	0.842	1.805
-Transferring	0.282	0.123		1.326	0.926	1.899
-Eating	0.026	0.855		1.026	0.779	1.351
-Continence	-0.015	0.912		0.986	0.760	1.277
-Bathing	-0.111	0.350		0.895	0.708	1.130
-Dressing and undressing	0.002	0.989		1.002	0.760	1.321
**Community life disorders**	**-0.046**	**0.001**	**	**0.955**	**0.929**	**0.982**
-**Meal preparation**	**-0.146**	**0.011**	*	**0.864**	**0.772**	**0.967**
-**Daily living rhythm**	**-0.169**	**0.013**	*	**0.844**	**0.738**	**0.965**
-**Hygiene**	**-0.199**	**0.003**	**	**0.819**	**0.719**	**0.934**
-Finance	-0.097	0.077		0.908	0.816	1.011
-Medications	-0.117	0.065		0.889	0.785	1.007
-**Relationships**	**-0.249**	**0.001**	**	**0.780**	**0.677**	**0.899**
**Acts that interfere with social adaptation**	**-0.068**	**0.007**	**	**0.934**	**0.889**	**0.982**
-**Telephoning**	**-0.173**	**0.018**	*	**0.841**	**0.729**	**0.970**
-**Shopping**	**-0.168**	**0.008**	**	**0.845**	**0.747**	**0.957**
-Mode of transportation	-0.125	0.045		0.882	0.781	0.997
**Degree of daily living ability**	**-0.387**	**<0.001**	***	**0.679**	**0.578**	**0.798**
**Daily life disorder ≥4**	**-0.552**	**0.001**	**	**0.576**	**0.415**	**0.798**

*p < 0.05; **p < 0.01; ***p < 0.001.

B, estimated regression coefficient.

**Table 8:** Physical complications (to the extent that inpatient treatment is required) at 3.5 years after the date of the survey and years and days of hospitalization until the date of the survey.

Items	B	P value	Significance level	Hazard ratio	Lower limit	Upper limit
Water intoxication	-0.060	0.836		0.942	0.536	1.658
Recurrent ileus	0.045	0.906		1.046	0.491	2.230
Recurrent pneumonia	-0.396	0.434		0.673	0.249	1.817
Others	-0.189	0.498		0.828	0.479	1.430
With physical complications	0.086	0.686		1.090	0.718	1.654
**Number of hospitalization days until survey date**	**-0.002**	**<0.001**	***	**0.998**	**0.998**	**0.999**
**Number of years of hospitalization until survey date**	**-0.682**	**<0.001**	***	**0.505**	**0.420**	**0.609**

***p < 0.001.

Psychiatric symptoms: positive symptoms, conceptual disorganization, suspiciousness, hallucinatory behavior, excitement, uncooperativeness, unusual thought content, and total BPRS score.

- Behavioral disorders: impulsiveness, B-item total score, and resistance to stress.
- Life disorders (score ≥ 4): community life disorders, living rhythm, hygiene, financial management, medication adherence, interpersonal relationships, acts interfering with social adaptation, telephoning, shopping, overall daily living ability, and the total daily life disorder score.

Additionally, certain items—such as comprehensive psychopathology (within “Psychiatric symptoms”) and behaviors including habits, posture, and undressing (within B-items of “Behavioral disorders”)—were significantly associated with hospitalization at 1.5 or 2.5 years but not at 3.5 years. No sub-items within the “Physical complications” category were significantly associated with hospitalization status at any timepoint. In addition to the Cox regression analysis, we conducted multiple regression analysis using the total number of hospitalization days as the dependent variable to provide a continuous perspective on hospitalization duration. At 3.5 years, male sex (B = 199.57, p = 0.0408), psychiatric symptoms (B = 202.17, p = 0.0288), and life disorders (B = 255.88, p = 0.0072) were significantly associated with longer hospital stays. At 2.5 years, male sex was also significantly associated with prolonged hospitalization (B = 203.08, p = 0.0076). These findings reinforce the contribution of these variables to long-term inpatient care in forensic psychiatric settings.

## 4 Discussion

To the best of our knowledge, this is the first study to describe the characteristics of psychiatric patients following long-term hospitalization in MTS Act wards in Japan. We applied the proposed “severe and chronic” criteria — originally developed for Mental Health and Welfare Law wards — to a forensic population, with outcomes tracked over a 3.5-year period. Of the 228 eligible individuals hospitalized for at least 1.5 years as of January 10, 2014, data from 226 (99.1%) were analyzed, reflecting a high inclusion rate.

This study is novel in three key respects: (1) it represents the first application of the proposed “severe and chronic” criteria to a forensic psychiatric population, (2) it identifies individual components most predictive of continued hospitalization, and (3) it offers a longitudinal, nationwide analysis of outcomes in MTS Act wards.

Under the Mental Health and Welfare Law, a “severe and chronic” classification is based on illness severity at a defined reference point and includes the following components: “Psychiatric symptoms,” “Behavioral disorders,” “Life disorders,” and “Physical complications”. Although “Physical complications” were initially hypothesized to contribute to long-term hospitalization, they were excluded from the final criteria due to a lack of significant association.

### 4.1 Factors for long-term hospitalization

Our analysis indicated that key components of “Psychiatric symptoms” and “Life disorders” were significantly associated with the length of hospitalization, consistent with previous findings (**Table 4**; 21, 22). In contrast, the major components of “Behavioral disorders” and “Physical complications” — the latter ultimately excluded from the proposed criteria — did not show a strong association with hospitalization duration. However, a limited number of sub-items within “Behavioral disorders” were associated with long-term hospitalization. This approach revealed that male sex, psychiatric symptoms, and life disorders were significantly associated with longer hospitalization at 3.5 years, while male sex was also a significant factor at 2.5 years. These findings underscore the relevance of demographic and clinical predictors in explaining long-term institutionalization and suggest that certain associations, particularly those involving sex, may emerge more clearly when considering cumulative hospital stay rather than timepoint-specific discharge.

Importantly, analysis of “Psychiatric symptoms,” “Behavioral disorders,” “Life disorders,” and “Physical complications” (**Table 5**; **Supplementary Tables 1**, **2**) revealed that positive symptoms and total scores within “Psychiatric symptoms,” as well as community life disorder and acts interfering with social adaptation under “Life disorders,” were significantly associated with prolonged hospitalization. Previous studies have also identified associations between hospitalization duration and positive symptoms measured by the BPRS (23), anxiety, depressive mood, financial difficulties (within “Life disorders”), as well as age and sex (24, 25). With respect to “Behavioral disorders,” B items—particularly resistance to stress—were associated with longer hospitalization. Although prior research identified aggressive behavior (26) and substance use (27) as contributors to extended stays, these associations were not confirmed in the present analysis. Suicidal ideation, notably, was found to be significantly associated with shorter hospitalization in this study, similar findings have been found in previous studies, such as a Canadian study of patients with schizophrenia (28, 29). For instance, a recent study reported that patients admitted for serious overdose remained hospitalized longer due to heightened medical and psychiatric risk, underscoring contextual and diagnostic variability across samples (30). Regarding “Physical complications,” no significant association was observed in this cohort, likely due to the low prevalence (approximately 10%) of such conditions. This finding differs from earlier studies linking diabetes mellitus (30), electrolyte imbalance and hypotension (31), and cardiovascular and metabolic diseases (32) with longer hospital stays (33). However, water intoxication, ileus, and pneumonia are common physical complications in psychiatric hospitalized patients, and may be factors for poor prognosis. Polydipsia may identify a subset of schizophrenia patients whose enhanced stress reactivity contributes to their mental illness (34). Constipation is a common problem during clozapine treatment which can progress to full-blown ileus which can be fatal (35). Advanced age, underweight, smoking habit, use of atypical antipsychotics, and large doses of antipsychotics were risk factors for pneumonia that is a major cause of death in patients with schizophrenia (36). In the “severe and chronic” criteria, it is thought that there was no significant difference in “Physical complications” because there were few people who met the criteria, but if “Physical complications” are expanded to include milder cases, it is possible that they could become a factor in long-term hospitalization. Additionally, no significant associations with age or sex were observed in this analysis, which diverges from prior findings in general psychiatry settings. For instance, a study found that older age and male sex were independently linked to longer psychiatric stays, suggesting that demographic influences may operate differently in forensic versus civil psychiatric population (37). This may reflect differences in demographic composition between patients in Mental Health and Welfare Law wards and those in MTS Act wards.

### 4.2 Limitations of the present study

This study has several limitations that should be considered when interpreting the findings. First, the number of participants in MTS Act wards was limited compared to those in Mental Health and Welfare Law wards, which imposed a physical constraint on sample size. Second, the duration of hospitalization prior to the survey varied among participants, and this variability was not incorporated into the analysis. Third, although most participants were diagnosed with schizophrenia, individuals with other psychiatric conditions were also included. Fourth, only the primary psychiatric diagnosis was recorded; comorbidities such as personality disorders and alcohol or drug addiction were not captured.

Fifth, post-discharge factors—including family relationships, residential environment, and community reintegration—were not examined, limiting the scope of interpretation regarding real-world outcomes. Prior studies have highlighted the impact of family involvement, institutional characteristics, and system-level predictors of length of stay and readmission, suggesting that these unmeasured variables may have influenced the results. Sixth, multiple comparisons were not adjusted for, given the exploratory nature of the study and the large number of variables assessed. This decision was intended to facilitate hypothesis generation for future research. Seventh, socioeconomic status and educational attainment — both known to influence psychiatric hospitalization duration — were not included due to constraints imposed by the predefined parameters of the study, which were determined by a research group affiliated with the MHLW. Eighth, the findings may not be generalizable to patients under the Mental Health and Welfare Law due to differences in legal frameworks and ward structures. In addition, various sociodemographic and clinical differences between the analyzed groups also may limit generalization. This study was limitedly carried in forensic psychiatric facilities in Japan, and because healthcare systems and forensic psychiatric care systems vary from country to country, there may be limitations to the generalizability of these results to other countries and healthcare systems. Ninth, data collection and analysis were both conducted by entities under the jurisdiction of the MHLW, raising the potential for institutional bias. Tenth, the follow-up intervals of 1.5, 2.5, and 3.5 years reflect time elapsed since the study’s initiation and do not directly represent individual patient length of stay. Additionally, the multiple regression analysis could not be performed for the 1.5-year timepoint due to insufficient data. As a result, findings from this method primarily reflect outcomes at 2.5 and 3.5 years, and interpretations should be made within that context. However, tracking patients hospitalized for more than 1.5 years over an additional 3.5-year period enabled identification of individuals at risk for long-term hospitalization. Finally, this study did not assess facility-level variation in resources or clinical practices, which may significantly affect hospitalization duration; future research should employ multilevel models to account for such institutional factors.

In conclusion, by applying the proposed “severe and chronic” criteria to a forensic psychiatric population, this study identified significant associations between prolonged hospitalization and major components of “Psychiatric symptoms,” “Life disorders,” and selected sub-items of “Behavioral disorders”. These findings offer important implications for discharge planning, clinical prioritization, and policy development within forensic mental health services.

## Data Availability

The original contributions presented in the study are included in the article/**Supplementary Material**. Further inquiries can be directed to the corresponding author.
